# Weed suppression greatly increased by plant diversity in intensively managed grasslands: A continental‐scale experiment

**DOI:** 10.1111/1365-2664.12991

**Published:** 2017-09-27

**Authors:** John Connolly, Maria‐Teresa Sebastià, Laura Kirwan, John Anthony Finn, Rosa Llurba, Matthias Suter, Rosemary P. Collins, Claudio Porqueddu, Áslaug Helgadóttir, Ole H. Baadshaug, Gilles Bélanger, Alistair Black, Caroline Brophy, Jure Čop, Sigridur Dalmannsdóttir, Ignacio Delgado, Anjo Elgersma, Michael Fothergill, Bodil E. Frankow‐Lindberg, An Ghesquiere, Piotr Golinski, Philippe Grieu, Anne‐Maj Gustavsson, Mats Höglind, Olivier Huguenin‐Elie, Marit Jørgensen, Zydre Kadziuliene, Tor Lunnan, Paivi Nykanen‐Kurki, Angela Ribas, Friedhelm Taube, Ulrich Thumm, Alex De Vliegher, Andreas Lüscher

**Affiliations:** ^1^ School of Mathematics and Statistics University College Dublin Dublin 4 Ireland; ^2^ Laboratory ECOFUN Forest Sciences Centre of Catalonia (CTFC) Solsona Spain; ^3^ Group GAMES and Department of HBJ, ETSEA University of Lleida Lleida Spain; ^4^ Waterford Institute of Technology Waterford Ireland; ^5^ Teagasc Environment Research Centre Co. Wexford Ireland; ^6^ Agroscope, Forage Production and Grassland Systems Zurich Switzerland; ^7^ IBERS, Plas Gogerddan Aberystwyth University Wales UK; ^8^ CNR‐ISPAAM Sassari Italy; ^9^ Agricultural University of Iceland Reykjavík Iceland; ^10^ Faculty of Biosciences Norwegian University of Life Sciences Ås Norway; ^11^ Agriculture and Agri‐Food Canada Québec Canada; ^12^ Teagasc Beef Research Centre Co. Meath Ireland; ^13^ Department of Mathematics and Statistics Maynooth University County Kildare Ireland; ^14^ Faculty of Biotechnical University of Ljubljana Ljubljana Slovenia; ^15^ NIBIO – Norwegian Institute of Bioeconomy Research Tromsø Norway; ^16^ CITA‐DGA Zaragoza Spain; ^17^ Plant Sciences Group Wageningen University Wageningen The Netherlands; ^18^ Department of Crop Production Ecology Uppsala Sweden; ^19^ Institute for Agricultural and Fisheries Research (ILVO) Melle Belgium; ^20^ Department of Grassland and Natural Landscape Sciences Poznan University of Life Sciences Poznan Poland; ^21^ UMR ARCHE INRA‐ENSAT Castanet Tolosan France; ^22^ Department of Agricultural Research for Northern Sweden Section of Crop Science Swedish University of Agricultural Sciences Umeå Sweden; ^23^ NIBIO ‐ Norwegian Institute of Bioeconomy Research Saerheim, Klepp st Norway; ^24^ Institute of Agriculture Lithuanian Research Centre for Agriculture and Forestry Kedainiai Lithuania; ^25^ NIBIO ‐ Norwegian Institute of Bioeconomy Research Løken, Heggenes Norway; ^26^ MTT Agrifood Research Finland Ecological Production Mikkeli Finland; ^27^ CREAF Cerdanyola del Valles Spain; ^28^ Institute of Crop Science and Plant Breeding University of Kiel Kiel Germany; ^29^ Institute of Crop Sciences University of Hohenheim Germany; ^30^ Present address: POB 323, 6700 AH Wageningen The Netherlands

**Keywords:** agro‐ecology, evenness, forage swards, functional diversity, generalised diversity‐interactions, legume–grass, nitrogen acquisition, sustainable agriculture, temporal development, transgressive weed suppression

## Abstract

1. Grassland diversity can support sustainable intensification of grassland production through increased yields, reduced inputs and limited weed invasion. We report the effects of diversity on weed suppression from 3 years of a 31‐site continental‐scale field experiment.
2. At each site, 15 grassland communities comprising four monocultures and 11 four‐species mixtures based on a wide range of species' proportions were sown at two densities and managed by cutting. Forage species were selected according to two crossed functional traits, “method of nitrogen acquisition” and “pattern of temporal development”.
3. Across sites, years and sown densities, annual weed biomass in mixtures and monocultures was 0.5 and 2.0 t  DM ha^−1^ (7% and 33% of total biomass respectively). Over 95% of mixtures had weed biomass lower than the average of monocultures, and in two‐thirds of cases, lower than in the most suppressive monoculture (transgressive suppression). Suppression was significantly transgressive for 58% of site‐years. Transgressive suppression by mixtures was maintained across years, independent of site productivity.
4. Based on models, average weed biomass in mixture over the whole experiment was 52% less (95% confidence interval: 30%–75%) than in the most suppressive monoculture. Transgressive suppression of weed biomass was significant at each year across all mixtures and for each mixture.
5. Weed biomass was consistently low across all mixtures and years and was in some cases significantly but not largely different from that in the equiproportional mixture. The average variability (standard deviation) of annual weed biomass within a site was much lower for mixtures (0.42) than for monocultures (1.77).
6. *Synthesis and applications*. Weed invasion can be diminished through a combination of forage species selected for complementarity and persistence traits in systems designed to reduce reliance on fertiliser nitrogen. In this study, effects of diversity on weed suppression were consistently strong across mixtures varying widely in species' proportions and over time. The level of weed biomass did not vary greatly across mixtures varying widely in proportions of sown species. These diversity benefits in intensively managed grasslands are relevant for the sustainable intensification of agriculture and, importantly, are achievable through practical farm‐scale actions.

## Abstract 2

Weed invasion can be diminished through a combination of forage species selected for complementarity and persistence traits in systems designed to reduce reliance on fertiliser nitrogen. In this study, effects of diversity on weed suppression were consistently strong across mixtures varying widely in species' proportions and over time. The level of weed biomass did not vary greatly across mixtures varying widely in proportions of sown species. These diversity benefits in intensively managed grasslands are relevant for the sustainable intensification of agriculture and, importantly, are achievable through practical farm‐scale actions.

## 1 INTRODUCTION

Agroecosystems are challenged to increase agricultural production to meet an increased demand for food production (Lüscher, Mueller‐Harvey, Soussana, Rees, & Peyraud, 2014) while preserving environmental functions and adapting to climate change (Tubiello, Soussana, & Howden, 2007). Increased efficiency (e.g. “getting more from less”) in the use of natural resources will underpin sustainable intensification of food production (Godfray et al., 2010). Plant diversity potentially provides a substitute for many costly agricultural inputs (Isbell et al., 2017). Weed growth represents a major source of inefficiency, diverting scarce resources (nutrients, water, light and labour) and results in about one‐third of yield losses in major crops (Oerke & Dehne, 2004). Weed control also diverts scarce resources, and herbicides incur significant environmental and economic costs. In pastures, weeds can impair forage quantity and quality resulting in reduced animal production, and increases the need for reseeding with its consequent costs. Here, we focus on weed control as an important objective in the design of a sustainable grassland agroecosystem.

Agroecosystems involve management practices that ultimately aim to control the utilisation of water, nutrients and light and a key question is: can management of species diversity enhance weed control? Empirically, increased species diversity in grassland communities is consistently associated with much lower weed biomass (e.g. Maron & Marler, 2008; Sanderson, Brink, Stout, & Leah, 2013). In general, increased species diversity is expected to reduce the availability of resources to weeds through a more complete use of resources by resident species (Renne, Tracy, & Colonna, 2006). Several other factors have been associated with negative effects of increased grassland diversity on weed biomass, e.g. identity effects (Crawley, Brown, Heard, & Edwards, 1999), niche pre‐emption (Mwangi et al., 2007), richness and functional group composition of mixtures (Byun, Blois, & Brisson, 2013), resident root mass and soil nitrate concentrations (Fargione & Tilman, 2005), increased crowding and species richness in localised plant neighbourhoods (Kennedy et al., 2002). Exceptions also occur where diversity is not associated with decreased weed invasion, measured either as richness (Smith, Wilcox, Kelly, & Knapp, 2004) or evenness (Emery & Gross, 2007). Most of these examples are typically from manipulations of species richness in semi‐natural grasslands; nevertheless, diversity effects (DEs) on weeds have been reported in more intensively managed grasslands (Finn et al., 2013; Frankow‐Lindberg, 2012; Sanderson, Brink, Ruth, & Stout, 2012). However, given the widespread distribution of intensively managed grasslands, the topic of weed invasion in these systems needs wider investigation of the impact of diversity (species identity, sown richness and sown species' relative abundance) and how outcomes generalise across environments and over time.

Some species or combinations of species can be particularly effective at suppressing weed biomass (Suter, Hofer, & Lüscher, 2017). Membership of a particular plant functional group (Fargione, Brown, & Tilman, 2003; Prieur‐Richard, Lavorel, Dos Santos, & Grigulis, 2002) or the presence of specific plant functional traits (Goslee, Veith, Skinner, & Comas, 2013) may improve the capacity of a community to resist invasion by weed species. This strongly suggests that to enhance ecosystem function in a multi‐species community, the targeted selection of species to include specific traits or to maximise trait diversity may be as, if not more, important than species richness per se (Suter et al., 2017). Specific traits that are expected to be important (in both yield production and weed suppression) include: (1) high yield potential, (2) capacity to achieve complementarity in nitrogen acquisition and utilisation (Nyfeler, Huguenin‐Elie, Suter, Frossard, & Lüscher, 2011) and (3) temporal differences in the development of species to improve early establishment (Tracy & Sanderson, 2004) and maintain interspecific interactions over time (Husse, Huguenin‐Elie, Buchmann, & Lüscher, 2016).

Legume‐based grasslands offer numerous agronomic and environmental advantages (Lüscher et al., 2014). In multi‐species mixtures that include legumes, nitrogen (N) resources are more efficiently used (Suter et al., 2015) and yield can be increased (Finn et al., 2013; Nyfeler et al., 2009). This is due to complementary acquisition of N sources (access to atmospheric N_2_ through biological fixation as well as available soil N) and to a lesser extent facilitation through N transfer from legumes to non‐legume species (Nyfeler et al., 2011). Ideally, a designed agro‐ecological system should also provide persistent and consistent weed suppression over temporal and spatial scales, over diversity gradients, and should be easily implemented at farm scale. Weed suppression would ideally be transgressive, i.e. weed biomass in mixture should be lower than in the most suppressive monoculture.

Previously, Finn et al. (2013) reported the results from 3 years of a 31‐site field experiment (Kirwan et al., 2014) that used four grassland species varying in two main traits, N acquisition and pattern of temporal development. They showed that total and sown species above‐ground biomass for four‐species mixtures were greater than in monocultures and summarised the value of mixtures in suppressing weeds compared with the average and best monoculture. Here, we analyse weed biomass from the same experiment in detail; we compare the effectiveness of sown species with different functional traits in weed biomass suppression in mixture compared to in monoculture; we explore the variation in weed suppression in mixtures across a range of sown evenness and along varying levels of functional traits in the mixtures. We note that exotic species were not a problem in monoculture or mixture at any of the sites. Using data from the 31‐site Agrodiversity field experiment (Kirwan et al., 2014), we address the following main questions, generalising across years and sites where possible:

1. Do grassland species in monoculture differ in their suppression of weed biomass?
2. Do mixtures transgressively suppress weed biomass?
3. To what extent is weed suppression in mixtures affected by differences in species' relative abundance?
4. Is weed biomass less variable in mixtures than in monocultures?

We show that four‐species grass–legume communities using species selected on the basis of functional traits “method of nitrogen acquisition” and “pattern of temporal development” can control weed biomass better than monocultures. Across 31 sites, weed biomass in mixtures was generally much lower than in monoculture communities for each of 3 years. On average, weed biomass in mixture was reduced by 52% relative to weed biomass in the most suppressive monoculture. On average, weed biomass in mixtures was maintained at relatively low levels across a range of mixtures varying considerably in sown evenness and across time.

## 2 MATERIALS AND METHODS

### 2.1 Experimental design

At each of 31 sites (30 European and 1 Canadian), 15 grassland communities comprising 4 monocultures and 11 four‐species mixtures of four forage species were sown at two seed densities (Table S1.1 in Appendix S1; Kirwan et al. (2014 **)** for full details of species used, sowing and management). There were 30 experimental plots per site, and data from 930 plots were analysed.

The four species selected at each site represent four distinct functional types based on combining two functional traits, “method of nitrogen acquisition” (Nyfeler et al., 2011) and “pattern of temporal development” (Finn et al., 2013). Functional types were: fast‐establishing, N_2_‐fixing legume (L_F_); fast‐establishing, non‐N_2_‐fixing grass (G_F_); temporally persistent, N_2_‐fixing legume (L_P_); and temporally persistent, non‐N_2_‐fixing grass (G_P_). A total of 11 locally adapted species represented the functional types across all 31 sites (Table S1.2 in Appendix S1). At a site, the four monocultures consisted of one of each of G_F_, G_P_, L_F_ or L_P_, and 11 mixtures were established by systematically varying sown species' proportions of these four species (Table S1.2 in Appendix S1). This resulted in four mixed communities dominated in turn by each species (sown 70% of one species and 10% of each of the other three species), six communities dominated in turn by pairs of species (40% of each species in the pair and 10% of each of the other two species), and an equiproportional community with 25% of each species. All 15 communities were sown at two densities; the high level was determined by local practice at the site and the low level was 60% of the high level. During the years of the experiments, plots were not weeded. The first year of data analysed was based on the first whole production year after the year of sowing. The biomass (t DM ha^−1^) of each sown species and weeds was calculated annually for each plot. In monocultures, biomass from species in the sown species pool other than the sown monoculture was included in weed biomass. Plots were surrounded by guard rows to inhibit invasion from adjacent plots (Kirwan et al., 2014). In some of our systems, we can find both exotic and non‐exotic invaders. However, the identification and quantification of individual invading species was not part of our study and therefore no precise reference to “exotic” can be made.

### 2.2 Analysis

We first summarised information on the proportion and biomass of weeds in mixtures and monocultures for each of 3 years and on average across years, and the extent of weed suppression in mixture compared with monocultures. At each site, we tested for transgressive suppression (Question 2) using a permutation test (Kirwan et al., 2007). To address Questions 1–4, we used the models below.

#### 2.2.1 Modelling weed suppression

For Questions 1–3, we used the generalised diversity‐interactions (GDI) modelling approach (Connolly et al., 2013). A model of weed biomass (*y*) in a community for a particular site and year is (Appendix S2.1):

(M0) $$y=\sum_{i=1}^{4}\beta_{i}P_{i}+\alpha A+\delta E_{\theta }+\varepsilon$$

Here *P*_*i*_ is the sown proportion of the *i*th species in the community (where *P*_*i*_ = 0 if the species is not included) and *A* is density (*A* = 0 for low and 1 for high density). β_*i*_ is the expected weed biomass of the monoculture of the *i*th species (*P*_*i = *_1) at the low level of sown density and α is the effect of density. In mixtures, $\begin{array}{c} \sum_{i=1}^{4}\beta_{i}P_{i} \end{array}$ gives the expected weed biomass in mixture (at low density) based solely on monoculture performances of the four species. In the basic model M0, the potential of all the pairwise interactions between any two species to contribute to function is measured by δ. This contribution of all pairwise interactions depends on the sown proportions of all species in the community, and in model M0, is δ*E*_θ_, called the DE for the community. The variable *E*_θ_ is a measure of the evenness of the community based on sown proportions of species (Appendix S3) and has a value of 0 for a monoculture and 1 for the equiproportional mixture. The coefficient θ allows a very wide range of forms for the DE and for the biodiversity–ecosystem–function relationship (Connolly et al., 2013).

**Figure 1:**
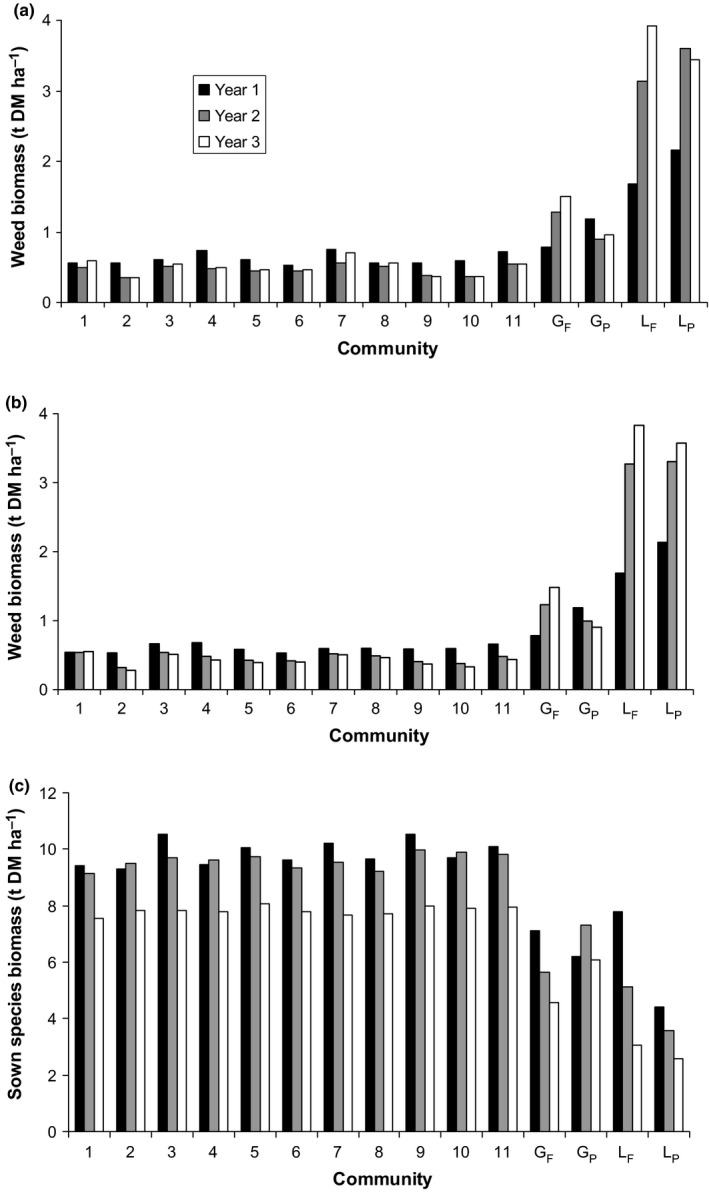
Annual weed biomass and sown species biomass (t DM ha^−1^) for each mixture (1–11 ordered according to Table S1.2 in Appendix S1) and for each monoculture (G_F_, G_P_, L_F_ and L_P_) for each of 3 years; (a) raw weed biomass averaged over sown densities and sites, (b) weed biomass values predicted from model M1 and (c) raw sown biomass averaged over sown densities and sites

There are many directions in which this model (M0) can be extended (Connolly et al., 2013; Kirwan et al., 2009) but the data summary of the mean weed biomass for all 15 communities for each year (Figure 1a) guided the choice (Tables S2.2 and S2.3 in Appendix S2.1). The level of average weed biomass was generally low across all 11 mixtures in each year, and weed biomass was generally much greater in monocultures, particularly in legumes. The greater weed biomass in legume compared with grass monocultures suggested that the effects of diversity in the model should be asymmetric, greater for mixtures with high sown legume content to reduce the mean weed biomass to the generally low weed levels in mixtures (Figure 1a). These two insights suggested a DE with a strong average suppression of weed biomass modified by the proportion of legumes, and potentially, also by the proportion of persistent species in a community. This led to a generalisation of M0 to include variables defining two functional axes, Grass–Legume (G‐L) and Fast–Persistent (F‐P).

(M1) $$y=\sum_{i=1}^{4}\beta_{i}P_{i}+\alpha A+\delta E_{\theta }+\delta_{L}L_{a}E_{\theta }+\delta_{P}P_{a}E_{\theta }+\varepsilon$$

In model M1, the DE now includes the evenness variable *E*_θ_ of M0 and two variables based on the sown proportions of legumes (*L*) and persistent species (*P*). The variables *L*_*a*_ *= L−*0.5 and *P*_*a*_ *= P−*0.5 represent the G‐L and F‐P functional axes respectively, and are both centred to be zero for the equiproportional community. β_*i*_ and α coefficients are interpreted as in M0. The expected DE for a community is $\text{DE}\;=\;\left(\delta +\delta_{L}L_{a}+\delta_{P}P_{a}\right)E_{\theta }$ and DE* = *δ for the equiproportional mixture.

Model M1 was fitted to annual weed biomass (t DM ha^−1^) (for details of all model fitting, model use for predictions and model selection, see Appendix S2.1). The estimate of θ (0.03) was first determined by profile likelihood (Pawitan, 2001) and all other fixed and random coefficients in M1 were estimated using a random coefficients (random across sites) mixed models maximum likelihood procedure with repeated measures analysis across years (Verbeke & Molenberghs, 2000). Various hypotheses were tested using predictions from the model and *t*, Wald and chi‐squared tests.

#### 2.2.2 Modelling weed biomass variation within a site

To address Question 4, we conducted a separate repeated measures analysis with community and site fixed to provide an estimate of the within‐site standard deviation of response for each community (see Table S2.4 in Appendix S2.2).

Analyses were mainly carried out using sas/stat software (9.3; SAS Institute Inc., Cary, NC, USA) and r (R Core Team, 2014).

## 3 RESULTS

### 3.1 Weed suppression varied among monocultures

**Figure 2:**
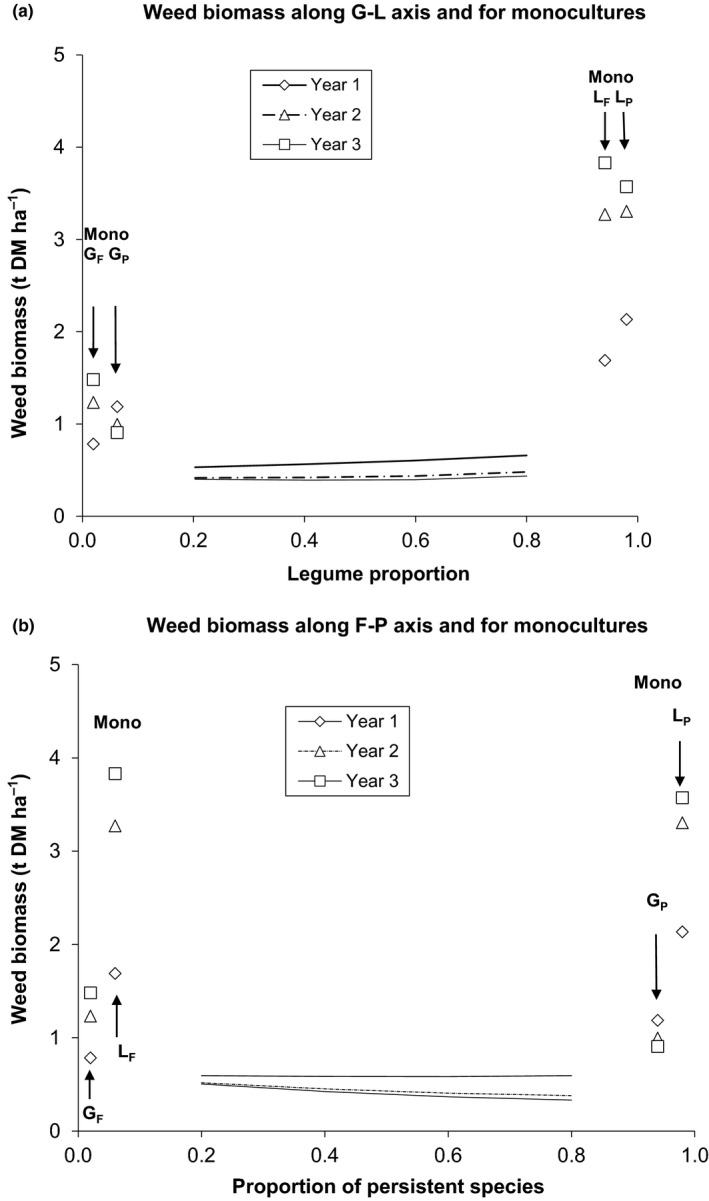
Effects of varying the ratio of (a) grass:legume and (b) fast:persistent functional traits on weed biomass. Weed biomass was predicted from model M1 for each monoculture community in each year. (a) Predicted weed biomass for mixtures based on a range of sown proportions of legumes lying between 0.2 and 0.8. Along this legume–grass axis, legume proportion (L) is equally composed of fast‐establishing (L_F_) and temporally persistent (L_P_) legume species and likewise for the two grass species (G_F_ and G_P_). (b) Predicted weed biomass for mixtures based on a range of sown proportions (P) of temporally persistent species lying between 0.2 and 0.8. Along this F‐P axis, P is equally composed of L_P_ and G_P_ and likewise with fast‐establishing species G_F_ and L_F_. Predictions for mixtures are made in the range L = 0.2–0.8 and P = 0.2–0.8 respectively, which is the range of sown legume (or sown persistent species) proportions in the design. Tests of significance of mixtures with monocultures are made for legume (and persistent species) inclusions rates of 0.2, 0.4, 0.6 and 0.8

There were marked differences between monocultures in weed suppression which changed across time (Question 1). In the first year after sowing (Year 1), annual weed biomass in monocultures (predicted from model) of the fast‐establishing grass G_F_ at average density (0.78 t DM ha^−1^) was less than that of the temporally persistent grass G_P_ (1.19 t DM ha^−1^; *p* = .005) but greater in the third year (*p* = .007; Table 1, Figure 2). While annual weed biomass for the temporally persistent grass (G_P_) monoculture did not vary significantly over years, the G_F_ monoculture had greater annual weed biomass in later years (*p* = .002, Year 1 vs. Year 3). Annual weed biomass in the monocultures of both legume types roughly doubled (*p* < .0001) between the first (average across legumes 1.91 t DM ha^−1^) and third (average 3.70 t DM ha^−1^) year. Annual weed biomass in the legume monocultures was about twice that of the grass monocultures in the first year (*p* < .01) but about three times (*p* < .0001) in the third year.

**Table 1:** The analysis of annual weed biomass for the first 3 years after sowing using model M1. Shown are estimates of coefficients (t DM ha^−1^), their standard errors (*SE*) and significance. The estimate of θ was 0.03 (*p* < .0001 compared with 1, Table S2.2, in Appendix S2.1) and the estimates for all other coefficients are for an average site

Coefficients	Effect of	Year 1			Year 2			Year 3		
		Estimate^a^	*SE*	*p*	Estimate	*SE*	*p*	Estimate	*SE*	*p*
β_*1*_	G_F_	0.78	0.124	<.0001	1.23	0.182	<.0001	1.48	0.193	<.0001
β_*2*_	G_P_	1.19	0.145	<.0001	0.99	0.210	<.0001	0.91	0.178	<.0001
β_*3*_	L_F_	1.69	0.160	<.0001	3.27	0.276	<.0001	3.83	0.295	<.0001
β_*4*_	L_P_	2.13	0.178	<.0001	3.30	0.290	<.0001	3.57	0.294	<.0001
α	Density	−0.12	0.028	<.0001	−0.04	0.027	.1368	−0.05	0.029	.0691
δ	*E*_θ_	−0.86	0.097	<.0001	−1.77	0.149	<.0001	−2.06	0.141	<.0001
δ_*L*_	L_a_ a *E*_θ_	−0.72	0.160	<.0001	−2.09	0.281	<.0001	−2.48	0.285	<.0001
δ_*P*_	P_a_ a *E*_θ_	−0.43	0.136	.0019	−0.13	0.241	.5922	0.13	0.221	.5575

a Estimates of monoculture effects are calculated at average density.

### 3.2 Transgressive weed suppression strong in mixtures

Across 31 sites and 3 years, predicted average weed biomass across all grass–legume mixtures (based on estimates of model M1 in Table 1) was 52% less than in G_P_, the most suppressive monoculture across years and sites (95% confidence interval: 30%–75% less). All mixtures showed transgressive suppression of weed biomass (*p* < .05) for each year of the experiment (Figure 1b; Question 2).

**Figure 3:**
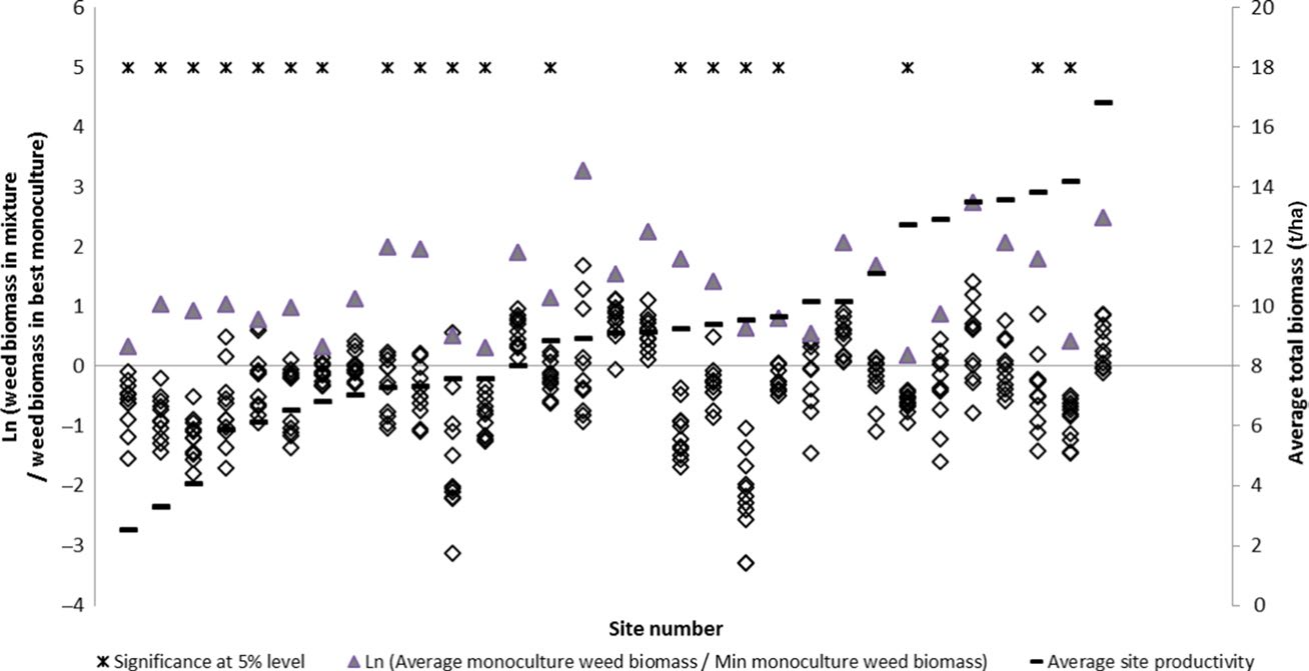
Levels of weed suppression and transgressive suppression by mixtures. Shown for each mixture and site is the natural log of the ratio of weed proportion in each mixture to weed proportion in the most suppressive monoculture on average across all years at the site (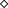). Each point represents one of the 11 mixtures and points below zero represent mixtures with lower weed proportion than the most suppressive monoculture (transgressive suppression). Sites are ordered by increasing average site productivity (see right‐hand axis). Significance of transgressive suppression at the 5% level (permutation test Kirwan et al., 2007) within a site is indicated by an asterisk. For each site log(average monoculture weed biomass relative to the weed biomass in the most suppressive monoculture) is also shown (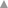) and each mixture (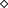) below (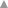) indicates weed suppression. (See Figure S1 in Appendix  for a similar figure for each year). Values are averaged over two sown densities [Colour figure can be viewed at wileyonlinelibrary.com]

At each site, weed biomass was suppressed in mixtures (Figure 3), being on average, 0.62, 0.46 and 0.44 t DM ha^−1^ in years 1–3 after sowing, respectively, compared with weed biomass in the most suppressive monoculture across all years (0.71, 0.62 and 0.70 t DM ha^−1^) and the average monoculture (1.45, 2.23 and 2.40 t DM ha^−1^) in those years (Table 2). Average weed proportion of total biomass was about 0.07 for mixtures and 0.33 for monocultures (Table 2). Across all years, weed biomass in mixture at a site was 25% of that in the average monoculture and 75% of that in the most suppressive monoculture for the site. Across years and sites virtually every mixture had a lower average weed biomass than the average of all sown monocultures (Table 3), and also when averaged over sites and years (Figure 1a). The reduction of weed biomass in mixtures was significantly transgressive in most sites and persisted across years (Table 3, Figure 3 and Figures S1.2 and S1.3 in Appendix S1). This result was independent of site productivity (Figure 3), which differed considerably between sites, with average annual total biomass ranging from about 3 to 18 t DM ha^−1^ year^−1^.

**Table 2:** Total annual biomass, annual weed biomass (both in t DM ha^−1^) and average weed proportion^a^ for mixtures and monocultures. Values are based on raw data averaged over the two sown densities and then averaged over sites for each year in the experiment

	Year 1	Year 2	Year 3
Total biomass			
Across all mixtures	10.48	10.18	8.24
Across all monocultures	7.83	7.72	6.38
Weed biomass			
Across all mixtures	0.62	0.46	0.44
In most suppressive monoculture^b^	0.71	0.62	0.70
Across all monocultures	1.45	2.23	2.40
Weed proportion			
Across all mixtures	0.07	0.06	0.08
In most suppressive monoculture	0.12	0.08	0.11
Across all monocultures	0.23	0.33	0.42

a Weed proportion = annual weed biomass/total annual biomass.

b Monoculture with lowest weed biomass averaged across all years at the site.

**Table 3:** Weed suppression in mixtures and sites. (a) Percentage of all mixtures across sites in which the weed biomass was lower than in the average monoculture (suppression) and than in the monoculture with lowest weed biomass at the site (transgressive suppression) for each of 3 years after sowing and averaged across years. (b) The number of sites showing suppression and significant transgressive suppression of weeds by mixtures (as measured by weed biomass) is shown for each year and across all years (using the nonparametric test in Kirwan et al. 2007). Results are based on raw data averaged over two sown densities at each site. See also Figure 3 and Figure S1.3 in Appendix S1

Year of harvest (number of sites)	(a) Mixtures		(b) Sites	
	Suppression (%)	Transgressive suppression (%)	Suppression	Significant transgressive suppression
All available years	99.7	67.4	31/31	19/31
Year 1 (31)	95.3	51.3	31/31	15/31
Year 2 (30)	99.7	64.8	30/30	17/30
Year 3 (24)	97.3	72.3	23/24	17/24

Transgressive suppression occurred along the G‐L and F‐P axes and the low level of weed biomass along these axes did not differ across years (Question 2). Weed biomass was predicted for four mixtures along the G‐L axis (Figure 2a) and the F‐P axis (Figure 2b). Predictions for mixtures were at proportions of 0.2, 0.4, 0.6 and 0.8 of L or P on the two axes respectively. Suppression was transgressive for predictions along both axes, significantly so in almost all cases (Figure 2) and was especially strong in mixtures dominated by legumes (Figure 2a).

### 3.3 Level of weed biomass consistently low across mixtures

The model showed that there were significant differences in weed biomass among the 11 mixtures, and several mixtures differed significantly from the equiproportional mixture in each year and overall. Yet the differences were not so great as to change the results relative to any monoculture. Relative to the equiproportional mixture as 100, the highest and lowest levels of weed biomass across all 11 mixtures were (highest, lowest), for years 1–3, (117, 91), (127, 75) and (141, 72) and overall (122, 81). Some patterns were evident in the differences among mixtures. Weed biomass was lower for communities dominated by grasses as opposed to legumes for each year, significantly so in year 1 (Figure 3a). Weed biomass was the same (year 1) or lower (*p* < .01, years 2 and 3) for communities dominated by persistent as opposed to fast‐establishing species (Figure 3b). In no case was there a significant difference between years for predictions along the G‐L or the F‐P axes (Figure 2).

### 3.4 Plot level variability of weed biomass lower in mixtures

**Figure 4:**
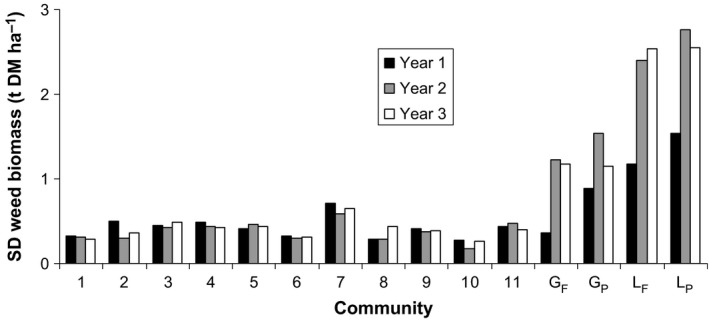
Standard deviation (*SD*) of annual weed biomass for a plot of each sown community for each of 3 years (estimate of within‐site replicate variation aggregated over sites). Community 5 is the equiproportional community and G_F_, G_P_, L_F_ and L_P_ are the monocultures

Not only was weed biomass much lower in mixtures but it was also much less variable. The estimated standard deviation (*SD* in t DM ha^−1^) of weed biomass for a plot within a site was, on average, lower (*p* < .0001) in mixtures (0.416) than in monocultures (1.770) (Figure 4, Table S2.4 in Appendix S2.2).

### 3.5 Generalised diversity‐interactions model

Model M1 fit the data very well (Figure 1b, Figure S2.2 in Appendix S2.1) and, in particular, showed the surprisingly flat patterns of weed biomass in mixtures apparent in the raw data (Figure 1a). Across all sites there were 11 species representing the four functional types (G_F_, G_P_, L_F_ and L_P_); however, model M1 with identity effects for four functional types fitted as well as a model with separate identity effects of the 11 species (see Table 2.3 in Appendix S2.1). Several other additional fixed terms examined did not add significantly to model M1 (Table S2.3 in Appendix S2.1). Thus, the GDI modelling approach (Connolly et al., 2013) provided an appropriate framework within which weed biomass could be predicted for specified proportions of constituent functional types and hypotheses could be tested.

## 4 DISCUSSION

### 4.1 Diversity enhanced weed suppression in grassland swards

Across 31 sites and 3 years, average weed biomass across all grass–legume mixtures was 52% less than in the most suppressive monoculture (95% confidence interval: 30%–75% less). Significant transgressive suppression of weeds was found in all mixtures. Weed biomass in mixtures was consistently low across a wide range of species' proportions for the duration of the experiment. Transgressive suppression of weed biomass was consistent across years, and was significant within most sites. Weed biomass was also less variable (standard deviation of plot weed biomass) in mixtures than monocultures. The suppressive effects of mixtures on weed biomass held over the wide range of environmental conditions (soil, climate and productivity) represented by the 31 experimental sites in Europe and Canada. We attribute the strong DEs on weed suppression to the targeted use of species with complementary functional traits for N acquisition and persistence.

### 4.2 Enhanced resource acquisition by mixtures largely explains weed suppression in mixtures vs. monocultures

In general, more diverse grasslands produce greater total and sown biomass as a consequence of diversity‐dependent processes that promote resource acquisition by the sward. A key question is whether increased acquisition of resources by grassland mixtures leads to reduced resource availability for weed growth. The extent of weed suppression can increase or decrease depending on whether dominant resident species either create a more competitive environment or alleviate stressful conditions for invaders (Smith et al., 2004). Our mixtures produced considerably more biomass than monocultures (Finn et al., 2013), and higher biomass production could be attributed to complementarity in functional traits leading to increased acquisition of resources (Hoekstra, Suter, Finn, Husse, & Lüscher, 2015; Suter et al., 2015). Taking biomass as a proxy for resource acquisition (in the absence of uptake studies), reduced weed biomass in mixtures implies that less resources were acquired by weeds in mixture than in monoculture, suggesting that this was a direct consequence of higher resource acquisition by sown species in mixtures. Despite the caveat that positive effects of diversity on total biomass can make it difficult to disentangle mechanisms leading to weed suppression (Tracy & Sanderson, 2004), we feel that it is useful to explore some mechanisms.

Given that N is often the most limiting resource in mesic grasslands, N acquisition may have an especially important influence on yields in most of our sites. Differences in N acquisition between monocultures and mixtures can affect soil N availability (as well as other resources; Hoekstra et al., 2015), with corresponding effects on weed biomass. For example, legume monocultures are prone to being invaded (Mwangi et al., 2007; Prieur‐Richard et al., 2002), partly by increasing N availability to invaders; in contrast, grass monocultures are generally more resistant to weed invasion (Mwangi et al., 2007), most probably related to their much bigger root mass (Hofer, Suter, Buchmann, & Lüscher, 2017) and stronger depletion of plant‐available soil N (Fargione et al., 2003; Hofer et al., 2017; Nyfeler et al., 2011). This is consistent with our results showing a greater weed biomass in the legume monocultures than in the grass monocultures. In contrast to grass monocultures, grass–legume mixtures have access to atmospheric N, which leads to greater sown biomass (Figure 1c; Lüscher et al., 2014; Suter et al., 2015). However, at levels of N fertiliser comparable to those used in our study, many grass–legume mixtures depleted the soil N as much or more than the grass monocultures (Nyfeler et al., 2011) and thus no facilitation of weed growth through the presence of N_2_‐fixing legumes in grass–legume mixtures must be inferred, in contrast to legume monocultures.

In addition to this strong role of N, more effective capture of light in mixtures than monocultures has been suggested as an important mechanism for weed suppression (Frankow‐Lindberg, 2012; Renne et al., 2006; Sanderson et al., 2012). Husse et al. (2016) showed that intensively managed monoculture or mixed grassland communities with >1.5 t DM ha^−1^ of yield per harvest captured >95% of incident light, leaving negligible light available at ground level for weed development. Yet, in less productive swards with less developed canopies, increased penetration of light could promote weed development at ground level. In our experiment, productivity varied considerably across sites (Finn et al., 2013) but on average the sown biomass per harvest of most mixtures and many monocultures exceeded 1.5 *t* ha^−1^. Therefore, in our productive swards, it is unlikely that light plays the key role in explaining transgressive suppression by mixtures; nitrogen acquisition and utilisation in swards are likely to be more dominant factors.

Although we explore the potential roles of N and light in understanding the suppressive effects of diversity on weeds, this does not necessarily exclude other mechanisms, e.g. weed species identity (Roscher, Temperton, Buchmann, & Schulze, 2009).

### 4.3 Weed biomass did not vary greatly across mixtures

The relatively small change in weed biomass across 11 mixtures, or when compared with the equiproportional mixture, is remarkable (Question 3, Figure 1b). Despite significant patterns in weed biomass among the mixtures, e.g. increasing suppression with increasing relative abundance in sown proportions of persistent species or grasses, the overall impression of these analyses is that weed biomass in mixture is reasonably robust to changes in species' relative proportions. This is important for theoretical and practical reasons. Any expectation that decreasing evenness might lead to a notable decline in weed biomass, and thence to a reduced suppressive effect relative to monocultures, was not realised here. The endpoints of the G‐L and F‐P gradients in Figure 2a,b, along which predictions of weed biomass were made, represent four mixtures used in the design, each of which contains 80% at sowing of grass, legume, fast‐establishing or persistent species, respectively. Each of these four mixtures were strongly suppressive relative to the closest monoculture species in the design. The suppressive effect of all mixtures relative to monoculture G_P_ (the measure of transgressive suppression here) remains roughly constant across time (Fig. 1b) but increases relative to all other monoculture species with time, mainly due to increased weed biomass in those monoculture species (see also Roscher et al., 2009). This shows that changing evenness is not a hugely influential force in these systems, either in respect of weed biomass relative to the equiproportional mixture or in respect of monocultures.

From an agronomic viewpoint, this relative unimportance of evenness in affecting weed biomass across mixtures means that there is no need to be over‐concerned with maintaining close limits on the relative abundance of species in the mixture; the desired outcome appears to be guaranteed irrespective of sown species' relative proportions. Indeed there is evidence that mixtures appear to be robust over time to some extreme changes in species' relative abundances; elsewhere we found apparent legacy effects of legumes in this experiment (Brophy et al., 2017).

We suggest that the mechanisms behind the relatively flat sown biomass response across mixtures and time are, as in the previous section on transgressive suppression, largely based on the process of N acquisition in grass–legume mixtures (Nyfeler et al., 2011). Differential light use by communities is even less likely to be a factor affecting weed biomass when we consider only mixtures. Our use of combinations of fast‐establishing and temporally persistent species was intended to maximise the interception of light in mixtures through quick gap‐filling during establishment of the grassland canopy and maintaining a largely closed canopy through its subsequent development. This light interception at all stages of growth ensured that for our productive mixtures, light was unlikely to contribute to variation in weed biomass across mixtures.

### 4.4 Agronomic relevance

We show that under a cutting management, weed invasion in grassland swards can be diminished through combining agronomic species selected for complementary traits regarding N acquisition and yield persistence in systems designed to reduce reliance on fertiliser N. Mixtures had consistently lower and less variable levels of weed biomass compared with monocultures across time, irrespective of species' proportions in the mixtures. Thus, grassland mixtures can sustain increased productivity (Finn et al., 2013) and persistently reduce weed biomass without being over‐concerned to manage the evenness of the species in the mixture. Furthermore, these results broadly apply across the continental span of our sites, which vary widely in agronomic conditions: annual rainfall (409–1,500 mm), annual mean air temperature (1.6–16.2°C) and annual applied N (0–150 kg/ha). These benefits provide further evidence for the multifunctional advantages of agronomic systems based on planned diversity (Dooley et al., 2015; Gaba et al., 2015; Lüscher et al., 2014).

## AUTHORS' CONTRIBUTIONS

J.C., M.T.S., A.L., J.A.F. and L.K. conceived the ideas and designed the methodology; all site co‐authors implemented the protocol and provided the data; J.C. and L.K. analysed the data; J.C., M.T.S., A.L., J.A.F., L.K., C.B., M.S. and R.L. led the writing of the manuscript. All authors contributed critically to the drafts and gave final approval for publication.

## DATA ACCESSIBILITY

The data are described in Kirwan et al. (2014) and are published in Ecological Archives at http://esapubs.org/archive, with accession number Ecological Archives E095-232.
