# Supplementary material for: Weed suppression greatly increased by plant diversity in intensively managed grasslands: A continental‐scale experiment
Source: J Appl Ecol. 2017 Sep 27;55(2):852–62. doi: 10.1111/1365-2664.12991 (PMC5836893; doi:10.1111/1365-2664.12991)
Supplement: Supplementary file 1 [file JPE-55-852-s001.doc]

**Supporting Information**

Appendix S1. Supplementary Figures and Tables and details of plot management.

Table S1.1. The sown proportions of four species in 15 communities. The first 11 are mixtures and the last 4 are monocultures. The two grasses are denoted G_F_ (fast-establishing grass) and G_P_ (temporally-persistent grass), and the two legumes L_F_ (fast-establishing legume), and L_P_ (temporally-persistent legume). Total proportion of grass (G) and fast-establishing species (F) is shown for each community. The high level of seed density was based on the local recommended sowing rate for each monoculture and the lower level of seed density was 60% that of the high level;

| Community | G_F_ | G_P_ | L_F_ | L_P_ | G | F | Evenness |
| --- | --- | --- | --- | --- | --- | --- | --- |
| 1 | 0.7 | 0.1 | 0.1 | 0.1 | 0.8 | 0.8 | 0.64 |
| 2 | 0.1 | 0.7 | 0.1 | 0.1 | 0.8 | 0.2 | 0.64 |
| 3 | 0.1 | 0.1 | 0.7 | 0.1 | 0.2 | 0.8 | 0.64 |
| 4 | 0.1 | 0.1 | 0.1 | 0.7 | 0.2 | 0.2 | 0.64 |
| 5 | 0.25 | 0.25 | 0.25 | 0.25 | 0.5 | 0.5 | 1 |
| 6 | 0.4 | 0.4 | 0.1 | 0.1 | 0.8 | 0.5 | 0.88 |
| 7 | 0.4 | 0.1 | 0.4 | 0.1 | 0.5 | 0.8 | 0.88 |
| 8 | 0.4 | 0.1 | 0.1 | 0.4 | 0.5 | 0.5 | 0.88 |
| 9 | 0.1 | 0.4 | 0.4 | 0.1 | 0.5 | 0.5 | 0.88 |
| 10 | 0.1 | 0.4 | 0.1 | 0.4 | 0.5 | 0.2 | 0.88 |
| 11 | 0.1 | 0.1 | 0.4 | 0.4 | 0.2 | 0.5 | 0.88 |
| 12 | 1 | 0 | 0 | 0 | 1 | 1 | 0 |
| 13 | 0 | 1 | 0 | 0 | 1 | 0 | 0 |
| 14 | 0 | 0 | 1 | 0 | 0 | 1 | 0 |
| 15 | 0 | 0 | 0 | 1 | 0 | 0 | 0 |

Table S1.2. Information on sites, choice of sown species, basic management and climate. Sites are distinguished by their identifier number in the database of Kirwa*n et a*l. (2014). Sites are arranged in order of declining total biomass of the best-performing monoculture over the three years.

| Site number in database | Country | Site | Latitude | Longitude | Altitude (m a.s.l.) | Species* | Nitrogen fertiliser (kg ha^-1^ per annum) | Harvests per annum  (year 1,2,3)^⊗^ | Size of plots (m^2^) | Annual rainfall (mm) | Annual mean temp (^o^C) | No. of years |
| --- | --- | --- | --- | --- | --- | --- | --- | --- | --- | --- | --- | --- |
| 1 | Belgium | Merelbeke | 50^o^59'N | 3^o^49E | 11 | Lp, Pp, Tp, Tr | 150 | 4,3,4 | 8.4 | 780 | 9.9 | 3 |
| 40 | Slovenia | Ljubljana | 46^o^3'N | 14^o^28'E | 300 | Lp, Dg, Tp, Tr | 120 | 4 | 8.6 | 1147 | 10.7 | 2 |
| 15 | Ireland | Wexford | 52^o^16'N | 6^o^30'W | 54 | Lp, Dg, Tp, Tr | 150 | 5 | 16.0 | 1033 | 10.1 | 3 |
| 21 | Netherlands | Wageningen | 51^o^58'N | 5^o^40'E | 7 | Lp, Dg, Tp, Tr | 0,108,108 | 5 | 6.0 | 760 | 9.6 | 3 |
| 10 | Germany | Renningen | 48^o^46’N | 9^o^11’E | 460 | Lp, Dg, Tp, Tr | 150 | 4,5,5 | 18.0 | 693 | 8.2 | 3 |
| 11 | Germany | St. Johann | 48^o^28'N | 9^o^18E | 700 | Lp, Dg, Tp, Tr | 150 | 4 | 18 | 1046 | 7.4 | 2 |
| 9 | France | Auzeville Tolosane | 43^o^05'N | 1^o^43'E | 162 | Lp, Dg, Tp, Ms | 120 | 3,2,3 | 6.0 | 680 | 13.0 | 3 |
| 34 | Switzerland | Zurich-Reckenholz** | 47^o^26'N | 8^o^32'E | 491 | Lp, Dg, Tp, Tr | 150 | 5 | 18.0 | 1031 | 9.4 | 3 |
| 24 | Norway | Ås | 59^o^40’N | 10^o^51’E | 95 | Lp, Dg, Tp, Tr | 135 | 3 | 12.0 | 785 | 5.3 | 3 |
| 35 | Wales | Aberystwyth | 52^o^26'N | 4^o^01'W | 30 | Lp, Dg, Tp, Tr | 90 | 4 | 6.0 | 1038 | 9.7 | 3 |
| 43 | Ireland | Athenry | 53^o^17'N | 8^o^44’W | 40 | Lp, Pp, Tr, Ta | 75 | 7 | 10 | 885 | 10.4 | 2 |
| 36 | Wales | Bronydd Mawr | 51^o^57'N | 3^o^37'W | 323 | Lp, Dg, Tp, Tr | 93 | 4,3,4 | 6.0 | 1500 | 8.2 | 3 |
| 27 | Poland | Brody | 52^o^26'N | 16^o^18'E | 91.4 | Lp, Dg, Tp, Tr | 90 | 4,3 | 9.0 | 587 | 8.0 | 2 |
| 44 | Ireland | Moorepark | 52^o^8'N | 8^o^16'W | 48 | Lp, Pp, Tr, Ta | 100 | 7 | 10 | 1207 | 9.5 | 2 |
| 26 | Poland | Brody | 52^o^26'N | 16^o^18'E | 94.2 | Lp, Dg, Tp, Tr | 120 | 4,4,3 | 9.0 | 587 | 8.0 | 3 |
| 22 | Norway | Saerheim | 58^o^46'N | 5^o^39'E | 90 | Lp, Dg, Tp, Tr | 0 | 3 | 12.0 | 1180 | 7.1 | 3 |
| 33 | Sweden | Öjebyn (Piteå) | 65^o^19'N | 21^o^24'E | 5 | Pp, Poa, Tp, Tr | 60 | 2,3,2 | 19.0 | 539 | 2.1 | 3 |
| 23 | Norway | Tromsø | 69^o^40'N | 18^o^56'E | 15 | Pp, Poa, Tp, Tr | 60 | 2 | 21.0 | 1031 | 3.1 | 3 |
| 19 | Lithuania (b) | Dotnuva | 55^o^24'N | 23^o^50'E | 71 | Lp, Dg, Tp, Tr | 120 | 3,2,2 | 6.5 | 650 | 6.1 | 3 |
| 7 | Finland | Mikkeli*** | 61^o^40'N | 27^o^13'E | 107 | Pp, Fa, Tp, Tr | 60 | 3 | 16.0 | 643 | 3.1 | 2 |
| 25 | Norway | Løken | 61^o^07'N | 9^o^04'E | 435 | Pp, Poa, Tp, Tr | 80 | 2 | 10.5 | 576 | 1.6 | 3 |
| 18 | Lithuania (a) | Dotnuva | 55^o^24'N | 23^o^50'E | 71 | Lp, Dg, Tp, Tr | 120 | 3 | 47.5 | 650 | 6.1 | 3 |
| 30 | Spain | Gosol | 42^o^13'N | 1^o^39'E | 1410 | Lp, Dg, Tp, Tr | 0 | 3 | 8.25 | 948 | 7.9 | 1 |
| 16 | Italy | Ottava | 40^o^44'N | 8^o^32'E | 80 | Lr, Dg, Mp, Ms | 31, 57, 61 | 4,5,5 | 9.0 | 547 | 16.2 | 3 |
| 20 | Lithuania (c) | Dotnuva | 55^o^24'N | 23^o^50'E | 71 | Lp, Dg, Tp, Tr | 120 | 3,3,2 | 24.0 | 650 | 6.1 | 3 |
| 52 | Canada | Lévis | 46^o^46'N | 71^o^12'W | 43 | Pp, Poa, Tp, Tr | 60 | 2 | 12 | 1175 | 5.26 | 3 |
| 31 | Sweden (a) | Svalöv | 55^o^55'N | 13^o^07'E | 55 | Lp, Dg, Tp, Tr | 0 | 3 | 8.8 | 700 | 7.7 | 3 |
| 32 | Sweden (b) | Svalöv | 55^o^55'N | 13^o^07'E | 55 | Lp, Dg, Tp, Tr | 0 | 3 | 8.8 | 700 | 7.7 | 3 |
| 13 | Iceland (a) | Korpa | 64^o^09'N | 21^o^45'W | 35 | Pp, Poa, Tp, Tr | 40 | 2 | 6.0 | 900 | 4.5 | 3 |
| 14 | Iceland (b) | Korpa | 64^o^09'N | 21^o^45'W | 35 | Pp, Poa, Tp, Tr | 80 | 2 | 10.0 | 900 | 4.5 | 3 |
| 28 | Spain | Zaragoza** | 41^o^44'N | 2^o^53'E | 225 | Lr, Dg, Mp, Ms | 61 | 2,3,1 | 9.0 | 409 | 14.3 | 3 |

*Species coded as follows. Non-fixing grasses: Dg *= Dactylis glomerata*, Fa = *Festuca arundinaca*, Lp = *Lolium perenne*, Lr = *Lolium rigidum*, Pp = *Phleum pratense,* Poa = *Poa pratensis.* N_2_-fixing legumes: Mp = *Medicago polymorpha*, Ms = *Medicago sativa*, Ta = *Trifolium ambiguum*, Tp *= Trifolium pratense*, Tr = *Trifolium repens*. Note that Lp for Lolium perenne here is not to be confused with L_P_ denoting persistent legume in text, tables and figures.

**The plots at Switzerland were weeded for the first two harvests in the establishment year and the plots at Spain-Zaragoza were weeded only in year 1 of harvesting.

***Barley was used on all plots as a nurse crop at establishment and harvested in the establishment year, as per conventional practice at this site

^⊗^ When only a single number is in this column this is the number of harvests in all years at the site.

**Plot management**

Plot management was carried out according to recommendations for agricultural grasslands under local conditions, which varied across sites (2 to 7 harvests year^-1^; 0 to 150 kg ha^-1^ year^-1^ of nitrogen fertiliser) but were always consistent across plots within a site (Table S1.1, Supporting Information). The duration of the experiment was three years for 24 sites, two years for 6 sites and one year for 1 site.


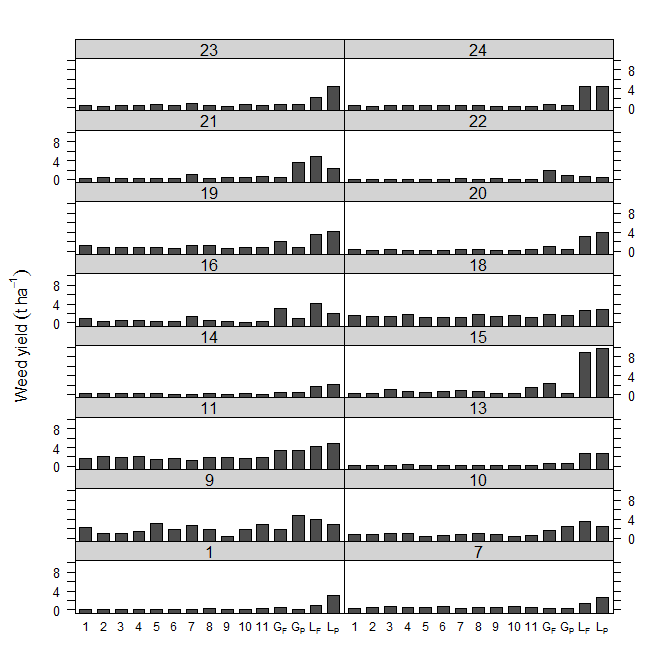

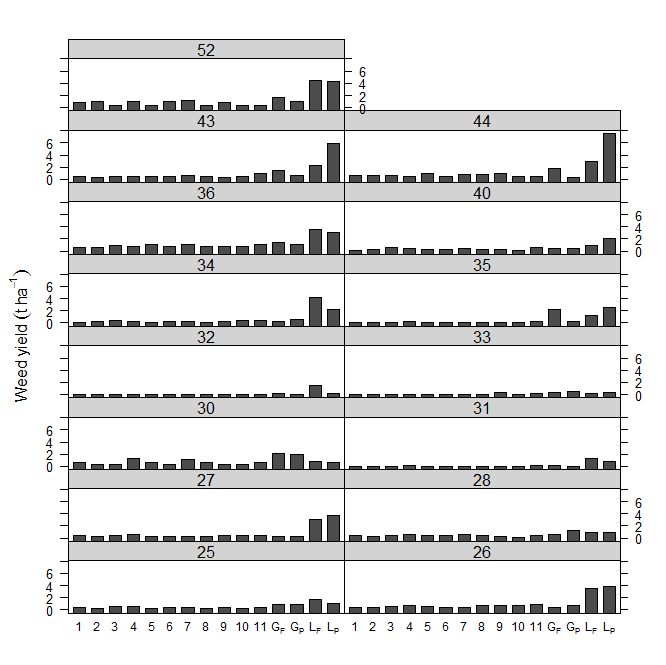


Figure S1.1. Mean annual weed biomass (t ha^-1^) for each community at each site averaged over time and sown seed density. Community 7 is the equiproportional mixture and communities 12 to 15 are the monocultures of G_F_, G_P_, L_F_ and L_P_.

Figure S1.2. Annual weed biomass (t DM ha^-1^) for average of sown monocultures (Av Mono), average of mixtures (Av Mix) and most suppressive monoculture (Best Mono) for each of 31 sites averaged across all experimental years. Source Unsown_species\across_sites_figure_b.xls


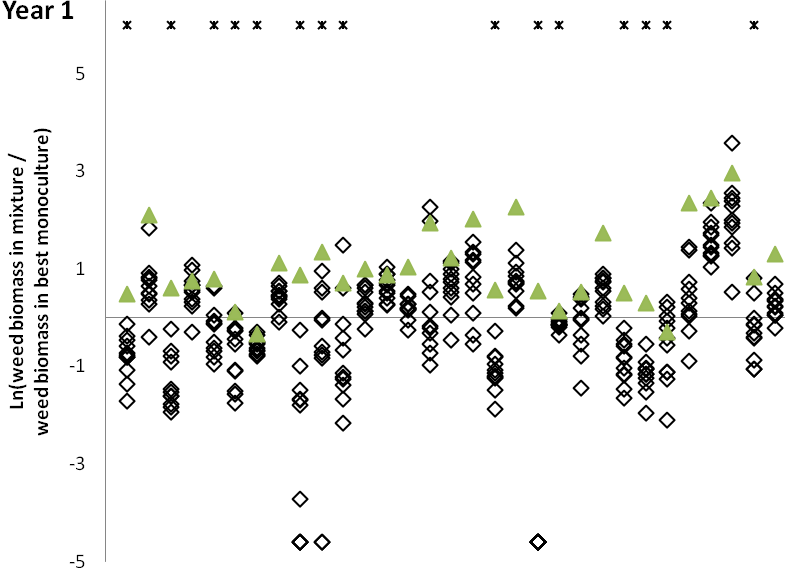

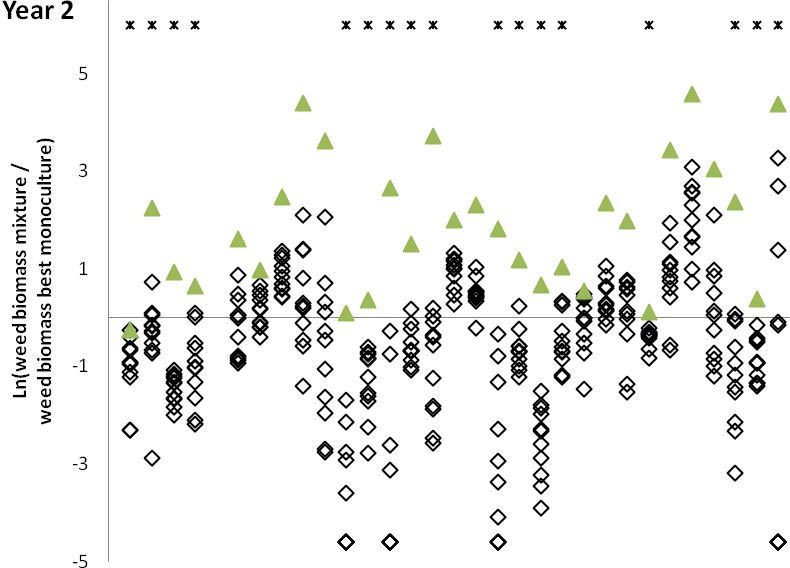

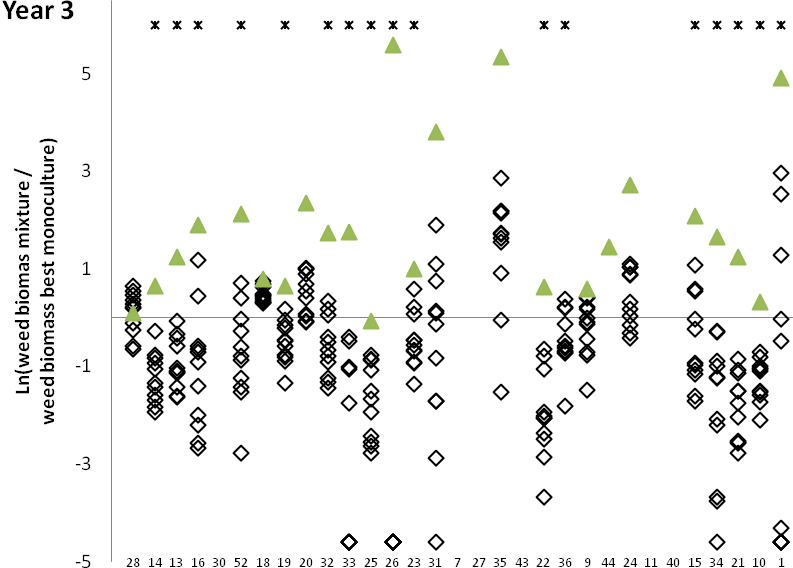


Figure S1.3. Transgressive weed suppression by mixtures. Shown for each mixture, site and year is the natural log of the ratio of weed proportion in each mixture to weed proportion in the best performing monoculture (**◇**). Each point represents one of the 11 mixtures and points below zero represent mixtures with lower weed proportion than the best performing monoculture (transgressive suppression). Sites are ordered by increasing average site productivity across all years. Significance of transgressive weed suppression within site at the 5% level is given by an asterisk. For each site log(average monoculture weed biomass relative to the weed biomass in the most suppressive monoculture) is also shown (▲). Values at -4.75 are for zero weed biomass in mixture.

**Appendix S2: Modelling strategy and results**

This section provides a description of the main models used in the analysis of the data to support the results presented in text.

***Appendix S2.1: Generalised Diversity-Interactions (GDI) model to analyse weed biomass***

The Generalised Diversity-Interactions model (Connoll*y et a*l. 2013) proposes the following description of weed biomass (*y*) in a 4-species pool. The model in a particular site is

$y=\sum_{i=1}^{4} \beta_{i}P_{i}+\alpha A+\sum_{i<j}^{4} \delta_{ij}(P_{i}P_{j})^{\theta}+\varepsilon$ (M)

(Note that M is a more general starting point model than M0 in the main text). Here *P_i_* is the proportion of the *i*th species in the community (= 0 if the species is not in the community). The term *A* indicates the level of sown density (coded 0 for low and 1 for high), the coefficient *β_i_* is the contribution to ecosystem function of the *i*th species when scaled by its relative abundance *P_i_*, and if *P_i_* = 1, *β_i_* estimates the weed biomass in monoculture of species *i* at low density. In a mixture, the sum of the coefficients scaled by their relative abundances (Σ*β_i_P_i_*) gives the expected response at low density based solely on monoculture performances. Interactions among species may also contribute to the response. The coefficient *δ_ij_* reflects the potential of species *i* and *j* to interact and the contribution of the interaction in a particular community to weed biomass is *δ_ij_(P_i_P_j_)*^θ^. The coefficient *θ* allows for flexibility in how species pairwise interactions contribute to ecosystem response and leads to a wide range of forms for the BEF relationship (Connoll*y et a*l. 2013). To make diversity coefficients more easily interpretable, in an experiment with s species in the species pool we rescale the term *(P_i_P_j_)^θ^* by using instead$I_{ij}=2\left(s^{2}P_{i}P_{j}\right)^{\theta}/s(s-1)$, which for a 4 species experiment is $I_{ij}=\left(16P_{i}P_{j}\right)^{\theta}/6$ (see rationale in Appendix S3). Where s = 4, the contribution to weed biomass from all the pairwise interactions in a community is called the diversity effect (*DE*), $DE = \sum_{\begin{matrix} i=1 \\ i<j \end{matrix}}^{4} \delta_{ij}I_{ij}$ and in a community in which sown proportions of all species are equal, the equiproportional community, this is $DE = \sum_{\begin{matrix} i=1 \\ i<j \end{matrix}}^{4} \delta_{ij}/6$, the average of the pairwise interaction coefficients.

Following Kirwa*n et a*l. (2009) and Connoll*y et a*l. (2013) the basic model above can be extended in several ways. For example, if all interactions have the same interaction potential, i.e. *δ_ij_* = *δ* for all pairs of species, this model becomes

$y=\sum_{i=1}^{4} \beta_{i}P_{i}+\alpha A+\delta E_{\theta}+\varepsilon$ M0

The diversity variable $E_{\theta}=\sum_{\begin{matrix} i,j \\ i<j \end{matrix}}^{4} I_{ij}$ is zero for a monoculture and 1 for the equiproportional community of all species in the species pool. (When *θ =* 1, *E_θ_* is the same as evenness as defined in Kirwa*n et a*l. (2007)). Coefficients may change over time and across sites.

In our data, the preliminary data summary in the main text motivates the extension of equation M that was finally selected (Fig. 1). Two features of the data stand out. Firstly, there was very little variation in weed biomass across all 11 mixtures in each year, while weed biomass was generally greater in monocultures, particularly in legumes. This stability in response across mixtures suggested a diversity effect that changed across mixtures but not in a simple symmetric manner (as would be the case with the quadratic diversity effect for θ = 1 (Kirwan *et al.* 2007) and also suggests a low value of *θ* (Connoll*y et a*l. 2013)). Secondly, the shape of the diversity effect clearly also depends on the proportions of the species present in the mixture; for example, the greater weed biomass in legume compared with grass monocultures suggests that the effects of diversity in the model should be greater for mixtures with high proportions of legumes (Fig. 1(a)). This led to a tentative generalisation of M0 to include variables defining two functional axes, Grass-Legume (G-L) and Fast-Persistent (F-P). This involved including the variables *L* = *P_LF_+P_LP_* and *P* = *P_LP_+P_GP_*, the total legume and persistent sown proportions respectively ( = 1-*G* and 1-*F*, where *G* and *F* are the total grass and fast-establishing proportions, respectively). Variables L and P were adjusted to be zero for the equiproportional community by defining *L_a_* = *L*-0.5 and *P_a_* = *P*-0.5 (to facilitate interpretation of the coefficients, see below) to extend model M0 as

$y=\sum_{i=1}^{4} \beta_{i}P_{i}+\alpha A+\delta E_{\vartheta}+\delta_{L}L_{a}E_{\theta}+\delta_{P}P_{a}E_{\theta}+\varepsilon$ M1

Here *A* is sown density. The diversity effect is $(\delta+\delta_{L}L_{a}+\delta_{P}P_{a})E_{\theta}$. For the equiproportional community *L_a_* and *P_a_* are zero and *E_θ =_* 1 and the value of the diversity effect is *δ*. The components *δ_L_* and *δ_P_* show how the two functional trait axes G-L and F-P contribute to the diversity effect for mixtures where sown species composition is not equiproportional. For mixtures, change in the diversity effect where the G-L proportions change but the F-P proportions are constant is measured by changes in $(\delta+\delta_{L}L_{a})E_{\theta}$. Similarly change along the F-P axis when G-L proportions are fixed is measured by changes in $(\delta+\delta_{P}P_{a})E_{\theta}$; if *δ_L_* and *δ_P_* are both zero the diversity effect is *δE_θ_* and is determined by change in *E_θ_* only. The prediction of ecosystem function (*y*) for any community is made from the full model M1.

There are two aspects of this model when *θ* is very small that are very relevant to the current data.

1. *E_θ_* is very stable across a very wide range of four-species communities for small *θ*. For example, for *θ =* 0.03 (as in the current data), *E_θ_* reduces from 1 at the equiproportional community to just 0.94 for a community dominated 95% by a single species, or to 0.97 for a community co-dominated (95%) by two species (Table S2.1). This means that change in the diversity effect across mixtures may be less sensitive to changes in sown evenness and more sensitive to changes in the overall legume and persistent species proportions. The diversity effect is approximately linear in *L_a_* and *P_a_*.
2. *E_θ_* tends to zero as the community approaches monoculture – i.e. as the sown species proportion of one species is close to 1. This means that the model is internally consistent, the diversity effect, which is a multiple of *E_θ_* tends to zero as the sown community becomes close to a monoculture.

Considering these two points we prefer the GDI model to a simple linear expression of the diversity effect in terms of *L_a_* and *P_a_*, as it is retains much of the simplicity of the linear interpretation just mentioned but is more coherent, since it incorporates the idea that the diversity effect must smoothly approach zero as communities are increasingly close to monoculture.

The Generalized Diversity-Interaction (GDI) modelling framework (Connoll*y et a*l. 2013) was able to mimic the surprising flat patterns of weed biomass apparent in the

raw data and provided an appropriate framework within which predictions could be made (e.g. along functional type axes) hypotheses could be tested.

***Table S2.1. Values of E_θ_ for a range of four-species communities for θ = 0.03. Also shown are the values of E, evenness as defined in Kirwan et al. (2007). P_i_ is the proportion of the ith species in the community. Rows in bold are rows in the design of the study (Table S1.2, Supporting Information).***

|  | *P_1_* | *P_2_* | *P_3_* | *P_4_* | *E_θ_* | *E* |
| --- | --- | --- | --- | --- | --- | --- |
| Various 4-species communities | | |  |  |  |  |
| **Design** | **0.1** | **0.1** | **0.1** | **0.7** | **0.975** | **0.640** |
| **Design** | **0.1** | **0.1** | **0.4** | **0.4** | **0.987** | **0.880** |
| **Design** | **0.25** | **0.25** | **0.25** | **0.25** | **1.000** | **1.000** |
|  |  |  |  |  |  |  |
|  | 0.1 | 0.1 | 0.1 | 0.7 | 0.975 | 0.640 |
|  | 0.1 | 0.1 | 0.3 | 0.5 | 0.986 | 0.853 |
|  | 0.1 | 0.1 | 0.5 | 0.3 | 0.986 | 0.853 |
|  | 0.1 | 0.1 | 0.7 | 0.1 | 0.975 | 0.640 |
|  | 0.1 | 0.2 | 0.1 | 0.6 | 0.983 | 0.773 |
|  | 0.1 | 0.3 | 0.1 | 0.5 | 0.986 | 0.853 |
|  | 0.1 | 0.3 | 0.3 | 0.3 | 0.995 | 0.960 |
|  | 0.1 | 0.3 | 0.5 | 0.1 | 0.986 | 0.853 |
|  | 0.1 | 0.5 | 0.1 | 0.3 | 0.986 | 0.853 |
|  | 0.1 | 0.5 | 0.3 | 0.1 | 0.986 | 0.853 |
|  | 0.1 | 0.7 | 0.1 | 0.1 | 0.975 | 0.640 |
|  | 0.3 | 0.1 | 0.1 | 0.5 | 0.986 | 0.853 |
|  | 0.3 | 0.1 | 0.3 | 0.3 | 0.995 | 0.960 |
|  | 0.3 | 0.1 | 0.5 | 0.1 | 0.986 | 0.853 |
|  | 0.3 | 0.3 | 0.1 | 0.3 | 0.995 | 0.960 |
|  | 0.3 | 0.3 | 0.3 | 0.1 | 0.995 | 0.960 |
|  | 0.3 | 0.1 | 0.1 | 0.5 | 0.986 | 0.853 |
|  | 0.3 | 0.1 | 0.3 | 0.3 | 0.995 | 0.960 |
|  | 0.3 | 0.1 | 0.5 | 0.1 | 0.986 | 0.853 |
|  | 0.3 | 0.3 | 0.1 | 0.3 | 0.995 | 0.960 |
|  | 0.3 | 0.3 | 0.3 | 0.1 | 0.995 | 0.960 |
|  | 0.3 | 0.3 | 0.1 | 0.3 | 0.995 | 0.960 |
|  | 0.3 | 0.3 | 0.3 | 0.1 | 0.995 | 0.960 |
|  | 0.3 | 0.5 | 0.1 | 0.1 | 0.986 | 0.853 |
|  | 0.5 | 0.1 | 0.1 | 0.3 | 0.986 | 0.853 |
|  | 0.5 | 0.1 | 0.2 | 0.2 | 0.990 | 0.880 |
|  | 0.5 | 0.1 | 0.3 | 0.1 | 0.986 | 0.853 |
|  | 0.5 | 0.3 | 0.1 | 0.1 | 0.986 | 0.853 |
|  | 0.7 | 0.1 | 0.1 | 0.1 | 0.975 | 0.640 |
| Extreme 4-species communities | | |  |  |  |  |
|  | 0.99 | 0.003 | 0.003 | 0.003 | 0.840 | 0.024 |
|  | 0.95 | 0.017 | 0.017 | 0.017 | 0.906 | 0.132 |
|  | 0.9 | 0.033 | 0.033 | 0.033 | 0.932 | 0.246 |
|  | 0.497 | 0.497 | 0.003 | 0.003 | 0.897 | 0.675 |
|  | 0.483 | 0.483 | 0.017 | 0.017 | 0.943 | 0.710 |
|  | 0.467 | 0.467 | 0.033 | 0.033 | 0.960 | 0.749 |
|  | 0.475 | 0.475 | 0.025 | 0.025 | 0.953 | 0.730 |
| Binary communities | |  |  |  |  |  |
|  | 0.99 | 0.01 | 0 | 0 | 0.158 | 0.026 |
|  | 0.95 | 0.05 | 0 | 0 | 0.165 | 0.127 |
|  | 0.9 | 0.1 | 0 | 0 | 0.168 | 0.240 |
|  | 0.8 | 0.2 | 0 | 0 | 0.171 | 0.427 |
|  | 0.7 | 0.3 | 0 | 0 | 0.173 | 0.560 |
|  | 0.6 | 0.4 | 0 | 0 | 0.174 | 0.640 |
|  | 0.5 | 0.5 | 0 | 0 | 0.174 | 0.667 |

***Modelling details and results***

There were four steps in modelling these data

- Selection of *θ*
- Selection of the best diversity model (fixed model)
- Selection of an appropriate random coefficients structure for model terms
- Selection of an appropriate repeated measures structure
- Additional tests on repeated measures structure using a separate model.

In the final model selected (M1) we used a value of 0.03 for *θ*, a GDI model that included coefficients (*β_i_*’s) representing identity effects of G_F_, G_P_, L_F_ and L_P_, a density effect (α) and the three diversity components (*δ*, *δ_L_*, *δ_P_*). These eight coefficients were assumed to be random across sites and to have different means and variances for each year. We also allowed for a random years within site effect to allow for year effects not captured in the random coefficient element of the model. The repeated measures part assumed unstructured covariance across years with a separate covariance structure for each community (*i.e.* the 15 communities listed in Table S1.2). We fitted this model using maximum likelihood (Pinheiro and Bates 2009).

There is inevitably some circularity in the modelling process – which comes first, the estimation of *θ*, the fixed model, the random structure or the repeated measures structure? Having found the model described above we subjected it to various tests to validate its selection. We checked (a) the estimate of *θ* for use with the final model using profile likelihood (Pawitan, 2001) (Table S2.2); (b) whether it was necessary to include the additional impact of separate identity effects for the 11 species constituting the four functional types; (c) whether a model with all 15 communities (the full reference model, Connolly *et al.* (2013)) and also their interaction with density as additional factors improved model fit; and (d) whether inclusions of the interactions between the coefficients in model M1 and density improved the model fit. Models were tested against each other using likelihood ratio tests (Table S2.3). Inference on the fixed terms of the fitted model M1 was based on Wald tests. There appeared to be considerable differences in the plot level variation (residual variation) of weed biomass across the 15 communities.

Alternative more complex random structures for the model were investigated (e.g. one that allowed covariance between coefficients within and between years) but we encountered convergence difficulties with these models and proceeded with the less complex variance components structure. Model fit was checked using tests between models and also through a graphical comparison of predicted means from M1 for each community with least squares means predicted under the variance assumptions of model M1 (Fig. S2.2).

A variant of M1 was fitted with the purpose of addressing question 4, on the variability of response within site for each community. We fitted a model whose fixed effects were those in the selected model M1 plus site (as a factor) and the interaction of site with each of the other terms in M1. This model had no random terms other than the repeated measures residual term. Various repeated measures structures were fitted using REML (Table S2.4) and compared using likelihood ratio tests. The repeated measures standard deviations displayed in Fig. 4 are drawn from the analysis of model M1.

*Estimation of θ.*

Table S2.2 shows how -2LL varied across a range of values of *θ*. The lowest value of -2LL was reached for *θ* = 0.03. The reference value *θ =* 1 (Connolly *et al.* 2013) gave a value of -2LL that was 129.4 greater than that for *θ* = 0.03, which is highly significantly different.

Table S2.2. Values of -2LL for values of *θ* varying from 0.01 to .04 and also including *θ =* 1.

| *θ* | Minus 2LL |
| --- | --- |
| *0* | 3829.0 |
| 0.01 | 3828.4 |
| 0.02 | 3828.2 |
| 0.03 | 3828.1 |
| 0.04 | 3828.3 |
| 1 | 3949.0 |

*The fixed model.*

Models M0 to M5 in Table S2.3 used the same random and repeated measure structure as described earlier. There was a significant diversity effect (M1 vs No Div, P < 0.0001); M1 fitted as well as models including a separate coefficient for each community in addition to density (M2 vs M1, P = 0.840) or a model (M3) adding the Community*Density*Year interaction (M3 vs M1, P = 0.306). Including identity effects of the 11 species comprising the four functional types did not improve the model fit (M4 vs M1, P = 0.598), nor did the addition of interactions of the fixed effects in M1 with density (M5 vs M1, P = 0.169). All the above were likelihood ratio tests between hierarchical models.

*The repeated measures structure*

The final model used a separate unstructured covariance matrix for plots of each community across years. There was considerable variation observed among the standard deviations across communities and time that suggested various hypotheses (Fig. 4). We attempted to fit several different versions of this repeated measures structure to facilitate tests of hypotheses about the standard deviations but encountered convergence problems. We adopted a different approach which is described below.

Table S2.3. Models fitted to weed biomass data for all three years of the experiment. Here Year is the year within the experiment. *E_θ_* is an interaction variable defined in text and its value is computed using θ = 0.03. A random coefficients model was used, with coefficients for identity effects, density effect and three diversity effects (see equation M1). Coefficients are assumed random across sites for each year with a separate variance component estimated for each coefficient. A variance component for Years random within site was also included. An unstructured repeated measures structure was assumed for plots across years, with a different covariance structure for each community. Tests of various hypotheses on fixed effects are shown as described in text. The same random and repeated measures structures were used in all models here and these models were fitted with maximum likelihood (ML). *L_a_* and *P_a_* are the scaled (centred) legume and persistent species proportions, respectively.

| Model | Fixed model. Terms in model are shown in parentheses [ ] | DF fixed | Number of random components | -2Log Likelihood |
| --- | --- | --- | --- | --- |
|  |  |  |  |  |
| No Div | No diversity effects [( G_F_ G_P_ L_F_ L_P_ Density)*Year] | 15 | 26 | 4039.5 |
| M1 | Final Diversity model [( G_F_ G_P_ L_F_ L_P_ Density E_θ_ LaE_θ_ PaE_θ_)*Year] | 24 | 26 | 3828.1 |
| M2 | Community model [(Community Density )*Year] | 48 | 26 | 3811.0 |
| M3 | Full community model [(Community Density Community*Density)*Year] | 90 | 26 | 3759.0 |
| M4 | Model with 11 separate species IDs [(Separate spp Density E_θ_ LaE_θ_ Pa E_θ_)*Year] | 43 | 26 | 3801.5 |
| M5 | M1 plus interactions of Density with effects in M1 [M1+ ( G_F_ G_P_ L_F_ L_P_ Density E_θ_ LaE_θ_ PaE_θ_)*Density*Year] | 42 | 26 | 3804.6 |
| Tests based on models | |  |  |  |
|  |  | Δdf | Δ(-2LL) | P |
|  | Test diversity (M1 vs No Div) | 9 | 211.4 | 0.000 |
|  | Test M1 against community model. (M2 vs M1) | 24 | 17.1 | 0.844 |
|  | Test effects of Full Community vs Community models (M3 vs M1) | 66 | 69.1 | 0.373 |
|  | Test 11 Separate spp ID vs 4 ID ( G_F_ G_P_ L_F_ L_P_) effects (M4 vs M1) | 19 | 26.6 | 0.114 |
|  | Test interactions of Density with both ID and diversity effects (M5 vs M1) | 18 | 23.5 | 0.172 |

Results Feb2015_revised Jan_2016_1 and in SAS program.

Figure S2.2. Weed biomass (t DM ha^-1^) predicted from model M1 for each of 15 communities plotted against weed biomass (t DM ha^-1^) averaged across all sites for each year. Averaging across sites used least squares means predicted from a model with the variance and repeated measures assumptions as in M1.. Source results_feb_2015_revised_jan_2016_3.xls

***Appendix S2.2: Model to test hypotheses about standard deviation within site***

In the GDI model above we tried to include separate unstructured repeated measures structure as a basis for hypothesis tests about the structure of residual variation but encountered convergence problems. Instead we fitted a model whose fixed effects were those in the selected model M1 plus site as a factor and the interaction of site with each of the other terms in the model, leaving just structure in the residual variation as random. In this model, various repeated measures structures were fitted using REML from which hypotheses about change in the standard deviation (SD) of response at the plot level could be tested. We examined whether the SD changed across mixtures, between mixtures and monocultures, between G and L monocultures, between monoculture grasses and monoculture legumes (Table S2.4).

Table S2.4. Comparison of various repeated measure covariance structures among communities. The form of covariance structures was separate variance component for each year. All models were fitted using REML and tests were chi-square between hierarchical models.

|  | Δ(df) | Δ(-2LL) | P |
| --- | --- | --- | --- |
| Between mixtures | 30 | 203.2 | <0.0001 |
| Between mixtures and monocultures | 3 | 556.7 | <0.0001 |
| Between G and L monocultures | 6 | 39.6 | <.0001 |
| Between monoculture grasses | 3 | 37.4 | <.0001 |
| Between monoculture legumes | 3 | 2.0 | 0.572 |

Source results Feb 2015_revised_jan_2016.xls rows 76 to 92

**Appendix S3:** Reformulation of the GDI model to give more ready interpretation of the diversity effect coefficients (δ, δ_ij_ etc.).

Suppose the Generalised Diversity-Interactions model for an s-species pool

$E(y)=\sum_{1}^{s} \beta_{i}P_{i}+\alpha M+\delta\sum_{i<j}^{s} \left(P_{i}P_{j}\right)^{\theta}$ MS1

Here, the diversity effect at the equiproportional mixture, where each *P_i_* = *1/s,* is and so is a function of the two coefficients *δ* and *θ*. It would be preferable to have a parametrization in which *δ* is the maximum diversity effect at the equiproportional mixture and *θ* reflects the shape of the interaction between species (discussed in detail in Connoll*y et a*l. (2013)). This can be achieved by using the variable $I_{ij}$in the diversity effect instead of $\left(P_{i}P_{j}\right)^{\theta}$ where $I_{ij}=2\left(s^{2}P_{i}P_{j}\right)^{\theta}/s(s-1)$. With this rescaling the value of the diversity effect is $\delta^{'}\sum_{i<j}^{s} I_{ij}$. At the equiproportional mixture, each *P_i_* = 1/s and so each $I_{ij}=2/(s(s-1))$ and $\sum_{i<j}^{s} I_{ij}$ = 1. Hence, at the equiproportional mixture, the diversity effect is simply *δ’* (which is the maximum diversity effect if *θ* < 1). So, *δ’* in the transformed model gives a simpler interpretation. The diversity effect for model MS1 may be rewritten as *δ’E_θ_* where $E_{\theta}=\sum_{i<j}^{s} I_{ij}$is zero for a monoculture and 1 for the equiproportional community of all species in the species pool. In M1 this version is used and the dash (‘) on the δ’ omitted as unnecessary notationally. For the more general model, in which separate pairwise interaction coefficients *δ_ij_* are fitted, the diversity effect at the equiproportional mixture becomes $\sum_{i<j}^{s} \delta_{ij}/(s(s-1)/2)$, the average pairwise interaction effect. The coefficient *θ* retains its original meaning. This change is easy to introduce at the programming stage whether one is using profile likelihood or nonlinear methods for the estimation of *θ*. An additional advantage of this reparametrization is that it will tend to reduce correlation between the estimates of the *θ* and *δ* coefficients.

**References for Supporting Information**

Connolly, J., Bell, T., Bolger, T., Brophy, C., Carnus, T., Finn, J.A., Kirwan, L., Isbell, F., Levine, J., Lüscher, A., Picasso, V., Roscher, C., Sebastia, M.T., Suter, M. & Weigelt, A. (2013) An improved model to predict the effects of changing biodiversity levels on ecosystem function. *Journal of Ecology*, **101**, 344–355.

Pawitan, Y. (2013) *In All Likelihood: Statistical Modelling and Inference Using Likelihood*. Oxford University Press. pp. 528.

Pinheiro, J.C. & Bates, D.M. (2009) *Mixed-effects Models in S and S-Plus*. Springer, New York, NY.

Kirwan, L., Lüscher, A., Sebastià, M.T., Finn, J.A., Collins, R.P., Porqueddu, C., Helgadottir, A., Baadshaug, O.H., Brophy, C., Coran, C., Dalmannsdóttir, S., Delgado, I., Elgersma, A., Fothergill, M., Frankow-Lindberg, B.E., Golinski, P., Grieu, P., Gustavsson, A. M., Höglind, M., Huguenin-Elie, O., Iliadis, C., Jørgensen, M., Kadziuliene, Z., Karyotis, T., Lunnan, T., Malengier, M., Maltoni, S., Meyer, V., Nyfeler, D., Nykanen-Kurki, P., Parente, J., Smit, H.J., Thumm, U. & Connolly, J. (2007) Evenness drives consistent diversity effects in intensive grassland systems across 28 European sites. *Journal of Ecology*, **95**, 530-539.

Kirwan, L., Connolly, J., Finn, J.A., Brophy, C., Lüscher, A., Nyfeler, D. & Sebastià, M.-T. (2009) Diversity–interaction modeling: estimating contributions of species identities and interactions to ecosystem function. *Ecology*, **90**, 2032–2038.

Kirwan, L., Connolly, J., Brophy, C., Baadshaug, O., Belanger, G., Black, A., Carnus, T., Collins, R., Čop, J., Delgado, I., De Vliegher, A., Elgersma, A., Frankow-Lindberg, B., Golinski, P., Grieu, P., Gustavsson, A.-M., Helgadóttir, Á., Höglind, M., Huguenin-Elie, O., Jørgensen, M., Kadžiulienė, Ž., Lunnan, T., Lüscher, A., Kurki, P., Porqueddu, C., Sebastia, M.-T., Thumm, U., Walmsley, D. & Finn, J. (2014) The Agrodiversity Experiment: three years of data from a multisite study in intensively managed grasslands. *Ecology*, **95**, 2680.
